# Peyronie's disease in the early phase: what to do?

**DOI:** 10.1590/1806-9282.20240078

**Published:** 2024-07-19

**Affiliations:** 1 Faculty of Medicine of São José do Rio Preto – São José do Rio Preto (SP), Brazil.; 2 Faculty of Medicine of São José do Rio Preto, Department of Urology – São José do Rio Preto (SP), Brazil.

Peyronie's disease (PD) is an abnormality characterized by fibrosis of the tunica albuginea that can be accompanied by pain, deformity, erectile dysfunction, discomfort, and/or dissatisfaction with one's self-image^1^. The prevalence ranges from 0.5 to 20.3%, but recent studies indicate underestimated data^1^.

In a study of guidelines for the diagnosis and treatment of PD of the American Urological Association (AUA), International Society for Sexual Medicine (ISSM), Canadian Urological Association (CUA), and European Association of Urology (EAU), Manka et al.^2^ reported that oral therapies present low level of evidence. Penile traction and intralesional injections are therapeutic options with unsatisfactory resullts^3^. There is consensus that the initial phase implies stability of the penile curvature for at least 3 months as well as a minimum period of 12 months without symptoms. Surgery should be reserved only after the stabilization of the disease.

Therefore, what should the clinical approach be during the first 12 months? This is a critical period in which many men deal not only with pain, erectile dysfunction, and deformity, the oral medicinal treatment of which is ineffective, but may also experience depression, low self-esteem, difficulty or inability having sexual relations, restrictions to intimacy, social isolation, and stigmatization^4^.

Considering the lack of standardization in the available literature on PD, much information used for therapeutic counseling of patients is based on a low level of evidence^1^. Thus, physicians face an ethical dilemma. Oral therapies are indicated without adequate scientific evidence, whereas patients deal with psychological and social issues concomitantly to the disease. One can say that both the physician and patient find themselves helpless in this period, despite the advances of current medicine.

Thus, there is a need for long-term clinical trials in the early phase of treatment. In the meantime, patients should be duly counseled on the risks and benefits of current therapies, highlighting sharing in the definition of the conduct with the physician and multidisciplinary team.
